# Themes in Train-the-Trainer Nutrition Education Interventions Targeting Middle School Students: A Systematic Review

**DOI:** 10.3390/nu13082749

**Published:** 2021-08-10

**Authors:** Christine St. Pierre, Win Guan, Leah Barry, Grace Dease, Sydney Gottlieb, Arielle Morris, Jamison Merrill, Jennifer M. Sacheck

**Affiliations:** 1 Milken Institute School of Public Health, The George Washington University, Washington, DC 20052, USA; gdease@gwu.edu (G.D.); jsacheck25@gwu.edu (J.M.S.); 2 Up2Us Sports, New York, NY 10018, USA; wguan@up2ussports.org (W.G.); jamisonmerrill@gmail.com (J.M.); 3 Department of Sociology, Tulane University, New Orleans, LA 70118, USA; lbarry2@tulane.edu (L.B.); sgottlieb@tulane.edu (S.G.); 4 School of Science & Engineering, Tulane University, New Orleans, LA 70118, USA; amorris12@tulane.edu

**Keywords:** nutrition education, middle school, train-the-trainer, peer-leaders, obesity prevention, systematic review

## Abstract

Context-appropriate nutrition education interventions targeting middle school students have the potential to promote healthy dietary patters that may help prevent unnecessary weight gain at a point in childhood development when youth experience increasing agency over their food choices. The aim of this review was to identify and synthesize themes in train-the-trainer approaches, intervention content and delivery, and youth receptivity across teacher, mentor, and peer-led nutrition education interventions that targeted middle school-age youth in urban, primarily low-income settings. A systematic, electronic literature search was conducted in seven electronic databases, PubMed/Medline, CINAHL, ERIC, PsycINFO, Scopus, SPORTDiscus, and Cochrane CENTRAL, using fixed inclusion and exclusion criteria. A total of 53 papers representing 39 unique interventions were selected for data extraction and quality assessment. A framework synthesis approach was used to organize the interventions into six categories and identify themes according to whether the intervention was classroom-based or out-of-school-based and whether adults, cross-age peers or same-age peers delivered the intervention. Ten of the interventions contained multiple components such that they were included in two of the categories. The review findings indicated that trainings should be interactive, include opportunities to role-play intervention scenarios and provide follow-up support throughout intervention delivery. Interventions targeting middle school youth should include positive messaging and empower youth to make healthy choices within their specific food environment context.

## 1. Introduction

The prevalence of childhood obesity in the United States is a major public health concern, particularly because obesity in youth often persists into adulthood and is associated with multiple chronic diseases, increased health care costs, and diminished quality of life [1,2]. Obesity prevalence is significantly higher among non-Hispanic black and Hispanic youth compared with non-Hispanic white and non-Hispanic Asian youth [3], and obesity prevalence tends to be higher in youth from households with lower head-of-household education levels and incomes [4]. Regular physical activity and healthy eating patterns are both important habits for obesity prevention, yet the vast majority of U.S. youth do not meet physical activity or fruit, vegetable, and water intake recommendations [5,6,7]. Historically under-resourced, urban neighborhoods in the U.S. have high concentrations of the populations identified as being at greatest risk for childhood obesity, and structural inequities in the built and food environments in these communities can make healthy choices even more difficult. Accordingly, obesity prevention interventions that target the physical activity and nutrition habits of at-risk youth have the potential to improve health outcomes in these communities for years to come.

Previous reviews that have examined the effectiveness of nutrition education interventions targeting youth, primarily in school-based settings, suggest the following components are important for successful interventions: behaviorally focused education, use of theoretical methods (e.g., skill building, self-assessment, social support, etc.), adequate dosage, and thorough training for those delivering the intervention [8,9,10,11,12]. Despite evidence for the importance of training and a reliance on those outside the nutrition profession—from classroom teachers to out-of-school program volunteers to older student peers—to deliver intervention programming, there is a lack of information on the most effective training methods for those delivering nutrition education interventions (e.g., training-the-trainer models). Furthermore, most reviews have lumped middle school age students with either elementary or high school students, rather than focusing specifically on strategies for targeting this age group as they are transitioning to adolescence and gaining increased agency over their food choices. The purpose of this review, therefore, was to build a larger evidence base for early adolescent nutrition education programs by identifying and synthesizing themes in training approaches, intervention content and delivery, and youth receptivity across teacher, mentor, and peer-led nutrition education interventions that targeted middle school-age youth in urban, primarily low-income settings.

## 2. Methods

This systematic review was conducted in accordance with the criteria set forth in the Preferred Reporting Items for Systematic Reviews and Meta-Analysis (PRISMA) [13]. While we searched the International prospective register of systematic reviews (PROSPERO) to ensure we were not duplicating a similar review, we did not register our protocol due to time constraints for finalizing our review and COVID-19 related processing delays at the time our protocol was developed. Primary research articles, protocol papers, and process evaluation papers were identified by searching PubMed/Medline, CINAHL, ERIC, PsycINFO, Scopus, SPORTDiscus, and Cochrane CENTRAL. The search strategy was developed by the research team and reviewed by a research librarian prior to initiation. An example of the search term strategy is provided in Supplementary Material Figure S1. The search was conducted in May 2020 and limited to English language results published between 2005 and 2020. The search was limited to results published in 2005 and later because it was in the middle part of the 2000s when the literature began to expand beyond raising the alarm about childhood obesity to the implementation and evaluation of health promotion interventions aimed at obesity prevention.

### 2.1. Inclusion and Exclusion Criteria

Given that our review question sought to identify and synthesize themes across the content and protocols of various nutrition interventions, we defined our inclusion criteria using the following PICo tool (Population, Interest, Context): Population targeted in the intervention should include at least part of the 10–14-year-old age range and should not be focused on youth with eating disorders or other specific nutrition needs related to a medical condition. Interest was interventions that were (1) nutrition, diet, or food-related and (2) included a description of the process for training those delivering the intervention or for training youth within the target population to deliver nutrition content to others. Context for the interventions were an urban school or community-based setting, either in the U.S. or in a setting comparable to a U.S. urban environment. Home-based interventions were excluded.

Qualitative studies related to the development or evaluation of an intervention were included if they described participatory research with youth from the target population or if interview subjects were those participating in or delivering the intervention and if there was at least one accompanying paper describing results of the intervention.

Given the challenges of following the same students consistently over a prolonged period of time in interventions that occur outside the classroom, papers were excluded if the intervention content was scaffolded and delivered over more than one academic year. Cross-sectional studies, review articles, book chapters, and poster abstracts were also excluded.

### 2.2. Secreening and Selection Process

Search results were uploaded to the Covidence online systematic review tool (Covidence, Melbourne, Australia), where duplicates were automatically removed by the review tool. A screening team consisting of four members worked in pairs so that two people independently screened the titles and abstracts of all articles identified through the search process and voted on inclusion or exclusion according to the criteria in the above section. The same process was followed in the full-text screening stage so that all papers were independently read and voted on for inclusion or exclusion by two screeners. At both stages, conflicts between screening team member votes were resolved by the first author. Two additional papers were added to the full-text review after the clinical trial registry for the study was identified through the systematic search process. Where multiple papers existed for the same intervention, all were included if the intervention met the inclusion criteria. Five additional papers were identified during the full-text stage that pertained to included interventions and were used in the data extraction stage.

### 2.3. Data Extraction and Synthesis Approach

Our focus on qualitative aspects of nutrition education interventions—namely train-the-trainer process and design, intervention content and delivery, and youth receptivity—led us to examine qualitative and mixed-methods approaches to synthesizing results. We sought to examine a wide variety of interventions—from those with a full nutrition education curriculum as in a classroom or a structured after-school program, to interventions that trained same-age peers to have informal conversations about nutrition, as well as scenarios in-between such as out-of-school settings led by community volunteers or young adult near-peers. We therefore set the inclusion criteria to allow for a diversity of methods, believing important themes would emerge among different types of interventions that would aid in developing new nutrition education programs with a robust training component. Based on our scoping searches, nutrition intervention expertise within the research team, and a review of qualitative synthesis methods, we selected a framework synthesis approach [14,15,16]. Framework synthesis is an application of the ‘framework analysis’ method used in primary research to systematic reviews [14]. It provides an *a priori* scaffold for organizing and mapping data from included studies, while permitting flexibility to iterate as the data is integrated into the framework [17].

In order to describe the elements of the nutrition education interventions identified through our search process consistently, we developed an analytical framework for grouping similar settings and training audiences, and then identified commonalities within each group. We wanted to examine classroom-based and extracurricular interventions separately to compare and contrast approaches in each of these settings. We also differentiated between populations trained to deliver the intervention to determine whether training approaches, intervention design or youth receptivity varied depending on whether adults, cross-age or near peers (older than target population but under age 23), or same-age peers delivered the intervention (see Table 1 below).

Once the categories A–F described above were identified, we organized the themes within each subgroup according to the following dimensions, driven by our review purpose stated previously: (1) train-the-trainer approaches, including number and duration of sessions and follow-up or support during the intervention; (2) common nutrition topics covered across the interventions; (3) format and delivery of the interventions, (4) youth receptivity, feedback, and outcomes measured; and (5) feedback and outcomes from those delivering the intervention, if assessed. All four members of the screening team extracted data independently and systematically for each of the categories and dimensions described above. The team members were once again partnered to independently extract data for each category or dimension. The lead author resolved any discrepancies in data extraction. Extracted data also included author information, funding source, study aims and location, demographic characteristics of participants, and theoretical basis for the intervention, if available.

### 2.4. Quality Assessment

Traditional methods of quality assessment used in systematic reviews do not always translate easily to qualitative reviews that include papers with a wide variety of study designs, as was the case for our review [14]. Our focus on training approaches, intervention messaging, and methods of evaluation rather than a primary emphasis on outcomes meant that the inclusion criteria allowed for a range of study types, from cluster randomized trials to protocol papers, that could not be directly compared to one another in terms of quality. While we determined that studies would not be excluded on the basis of quality due to their potential to provide information valuable to our review question, we assessed the methodological strengths and limitations of the included studies in order to consider the effect of limitations on our review findings [18]. For this assessment, we used a modified version the CASP tool for qualitative research [19]. We omitted the question asking whether qualitative methodology is appropriate, given that many of our included studies had quantitative research questions and were not designed to be assessed according to qualitative criteria. However, when the questions were applied according to the aim of each study, the tool provided a consistent framework for identifying methodological strengths and limitations. Without a definitive tool for such an assessment across a range of study types, the CASP tool was the best fit for our analysis.

We dichotomized studies into those with adequate methodological strength and those that were methodologically limited. The review team decided that studies with four or more ‘no’ responses using the CASP tool would be categorized as methodologically limited. Any differences in opinion in quality assessment were discussed among the review team until we reached consensus on categorization of each study. Following our review synthesis, we conducted a sensitivity analysis to determine whether the exclusion of methodologically limited studies affected the themes identified or the complexity of detail within each theme [19,20].

## 3. Results

**Figure 1:**
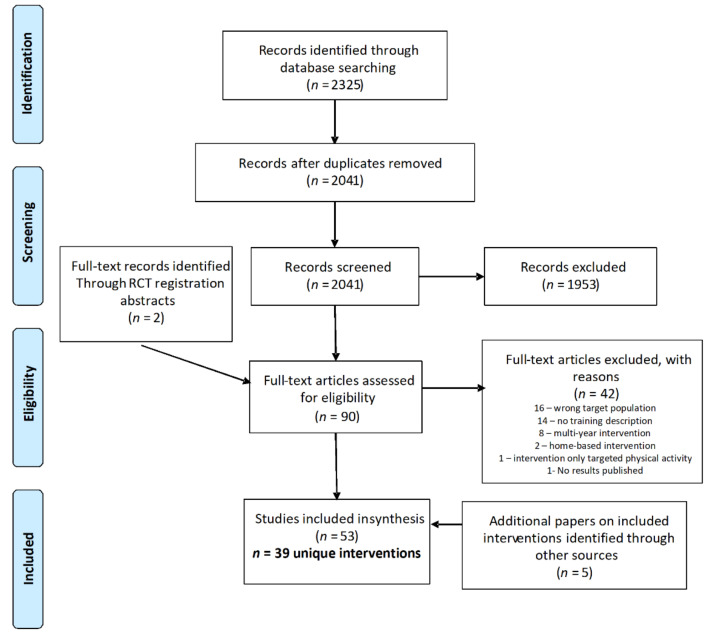
PRISMA flowchart of study selection process.

The database search yielded 2041 articles after duplicates were removed; following title and abstract screening, 88 papers were retained for full text screening. Two additional full text papers were added from hand searching after the intervention was identified in a clinical trial protocol captured by the database search. The screening process identified 48 papers meeting the criteria for inclusion in the review, representing 39 unique interventions. An additional five papers were identified during the full text review that pertained to included interventions, for a total of 53 papers used in the data extraction process [21,22,23,24,25,26,27,28,29,30,31,32,33,34,35,36,37,38,39,40,41,42,43,44,45,46,47,48,49,50,51,52,53,54,55,56,57,58,59,60,61,62,63,64,65,66,67,68,69,70,71,72,73]. The most common reasons for exclusion at the full text stage included a target population outside the 10–14-year-old age range (*n* = 16), lack of description of the training process (*n* = 14), and an intervention scaffolded over multiple school years (*n* = 8). The flowchart in Figure 1 details the screening and selection process.

Of the 39 interventions included, the majority (*n* = 23) took place in the United States. Interventions outside the U.S. were in Europe (*n* = 7), Canada (*n* = 2), China (*n* = 2), and one each in Australia, New Zealand, Aruba, and Ethiopia. The framework shown in Table 1 above was applied to each of the 39 interventions to divide them into categories. Eight of the interventions [21,40,45,52,53,59,65,70,72,73] included two different groups that were trained as facilitators, so they were included in both applicable categories. Two interventions [33,46,47] had both an in-class curriculum component and an out-of-school component where students interacted with professional athletes; these are included in both groups A and D. Five interventions trained the target population themselves to deliver the intervention to others. These were classified into the same age peer groups since the training was targeted at 10–14-year-olds. The trained youth then delivered nutrition information to the community (Group F) [45,48,55,63], or taught younger students (Group C) [67].

In our quality assessment, 34 of the 39 studies were categorized as having adequate methodological strength. We observed the following limitations most commonly across included studies: (1) study design, as not all interventions could be conducted in a randomized setting or include a comparison group, (2) insufficient power to detect an intervention effect, (3) short follow-up periods that do not allow for assessment of long-term behavior change, and (4) difficulty in obtaining reliable dietary intake information from participant self-report. We conducted a sensitivity analysis by excluding the five studies identified as methodologically limited [22,27,58,64,68] from the synthesis, and still identified the same themes. Although these studies made contributions toward our review question, such as incorporating the community into intervention design [22] or empowering youth to advocate in their community [58], these details were also present in other included studies. The “thickness” of the themes was not diminished when the sensitivity analysis was performed, as none of the details identified within each theme were dependent on only one study.

In the sections that follow, we identify the key themes from each of the intervention groups, and a summary table is provided for each group. We summarize the training design, intervention format and delivery, and the evaluation indicators used for each intervention, along with notable results. For outcome results, we focused on significant changes in food groups of concern for adolescents: fruit and vegetables, and sugar-sweetened beverages. We also summarized qualitative feedback from intervention participants and those delivering the intervention when it was reported in studies.

### 3.1. Group A: Classroom-Based Interventions That Trained Adults

Nutrition education interventions delivered in a classroom setting where adults were the population trained to deliver the intervention was the largest group in our categorization system (*n* = 19 unique interventions). Teachers were the primary population trained, though in some instances, graduate students, volunteers, or assistants were also trained as co-teachers [22,49,50,51,54,56,57,69]. In five of the interventions, the authors describe an initial training with follow-up trainings or regular meetings with teachers during the intervention to provide support or resolve any issues that arose [22,28,29,30,31,32,45,46,47,51]. Most trainings are described as in-services or professional development for teachers, and several note that trainings were interactive, giving teachers the opportunity to practice delivering the content to the trainers and their peers. Four interventions emphasized encouraging those delivering the intervention to tailor examples and references to the local context [22,45,49,50,51].

Basic nutrition concepts were covered across the interventions, e.g., energy balance, food groups, portion sizes, healthy meals, and snacks. Key messages included increasing fruit and vegetable consumption, decreasing sugar-sweetened beverage and unhealthy snack consumption, and eating out less often. Several of the interventions included activities where students learned to read nutrition labels, track their food intake, set goals, or make plans for healthy eating, assess their neighborhood food environment, and develop strategies for making healthy choices within their context.

In four of the interventions, the curriculum was incorporated into students’ PE class [21,22,46,47,72,73], while in two others, it was incorporated into science class and specifically mentioned an inquiry-based approach for student learning [27,28,29,30,31,32,33]. Eight of the interventions explicitly mentioned aligning the content with state or national standards or curriculums [27,28,29,30,31,32,34,36,37,49,50,51,64]. In three instances, homework was included in the intervention design that was meant to facilitate students engaging with their family about the nutrition information [34,37,49,50]. Four interventions also involved a connection to school meals, often engaging students in advocating for healthy meal options they would enjoy [45,54,56,57,69].

Evaluation measures varied by intervention but commonly included analysis of anthropometric changes, shifts in dietary intake, and differences in knowledge or attitudes between pre-and post-intervention. For process evaluation, six of the interventions included observations of a portion of the classroom lessons by research staff to assess fidelity and student engagement [21,28,29,30,31,32,45,61,69,72,73]. Four of the interventions held student focus groups post-intervention to gain qualitative feedback [22,27,54,69], and two used a student satisfaction survey [28,29,30,31,32,33]. Table 2 below further describes the interventions included in Group A.

### 3.2. Group B: Classroom-Based Interventions That Trained Cross-Age Peers

There were four interventions that trained cross-age peers to deliver classroom-based lessons. Three of the interventions trained high school students [21,35,38], and the remaining one trained undergraduate university students [59]. In three of the four interventions, cross-age peers taught as a group [38,59] or with teachers [19]. Two of the interventions had weekly training for cross-age peers [21,35], and two emphasized the opportunity to practice delivering the intervention as part of the training [21,38].

One of the interventions focused specifically on healthy beverages [59]; the other three all included both nutrition and physical activity content. In each of the interventions, there was an emphasis on games as a medium for delivering the nutrition content. Compared with the interventions in Group A, these interventions were delivered over a shorter period of time, except for the one where cross-age peers worked with PE teachers [21]. Role-modeling healthy habits was a key emphasis with cross-age peers.

Outcome measurers were similar to Group A, though one of the studies evaluated outcomes and intervention acceptability for the cross-age peers [38], which could serve as indicators of the engagement level of those delivering the intervention. Of note, for one intervention, only the group that received the intervention from cross-age and same-age peers showed a sustained significant decrease in SSB consumption [59]. The four interventions are described in Table 3.

### 3.3. Group C: Classroom-Based Intereventions That Trained Same-Age Peers

Two classroom-based interventions trained same-age peers as facilitators; in both cases the peers had strong support and mentorship from either adults or cross-age peers. Both interventions also put the same-age peers in groups to deliver the intervention material. In one of the interventions, those trained were in our target population age range and delivered the intervention to younger elementary students [67]. This intervention is included here and not in Group B in order to examine it alongside other trainings designed for the same age group. The training elements in these interventions provide insight into strategies for empowering the target age group, which is a key component of many nutrition education interventions for middle schoolers. Table 4 describes the interventions included in Group C.

### 3.4. Group D: Community, Afterschool, or Extracurricular Interventions That Trained Adults

Ten interventions trained adults to deliver nutrition education in a context outside the classroom: four were based in the community [33,46,47,58,62], four were afterschool programs [40,60,71,72,73], one was delivered in multiple settings that included both community and afterschool programs [52,53], and one took place during school lunch [70]. Four of the interventions also included classroom-based components [33,46,47,70,72,73].

The types of interventions varied widely, as did the training format, but a common theme across all the interventions in this group was an expansion beyond basic nutrition education. Four of the interventions particularly emphasized role models/mentors [33,46,47,58,70]; two of which included activities with professional sports teams in the community. Interventions in this group also went deeper into the food environment, food sources, student advocacy for healthy changes, and addressing barriers to healthy eating than did the interventions that were classroom- based. In terms of evaluation, two interventions used student surveys to measure acceptability [33,52,53], and two conducted focus groups with youth to assess barriers and facilitators to healthy eating [60,62]. Of note in these focus groups with youth in different parts of the U.S.: both groups expressed limited availability of healthy food in their home and neighborhood environments as a major limiting factor in healthy eating, especially when it came to fresh fruit and vegetables. The interventions included in Group D are presented in Table 5 below.

### 3.5. Group E: Community, Afterschool or Extracurricular Interventions That Trained Cross-Age Peers

Four interventions trained cross-age peers to implement interventions outside a classroom setting. All interventions trained high school students, though one intervention also trained undergraduate college students in their first wave [41,42,43,44]. Similar to classroom-based interventions using cross-age peers, facilitators taught as groups of leaders rather than as individuals. One intervention paired cross-age peers with adults with the goal of training them throughout the intervention to facilitate sessions on their own [40]. Another gave cross-age peers autonomy in scheduling intervention events with younger peers, with adult support available for planning and guidance [65]. Both of these interventions had less consistency in participation among cross-age peers than the one that had a more structured intervention delivery plan [41,42,43,44]. The fourth intervention in this group used cross-age peers to develop intervention content that was then used in a virtual format and accompanied lessons taught by adults [52,53]. This element of the intervention was noteworthy because it encouraged cross-age peers to tap into their interests, whether sports, ballet, art, etc., and connect that to nutrition content.

One of the interventions conducted post-intervention focus groups with both youth participants and cross-age peers [41,42,43,44]. Youth reported they particularly enjoyed the games and cooking lessons. Cross-age peers reported that discussions were the most difficult intervention element in which to engage youth. They noted that it would have been helpful to have more role-playing opportunities during the training to address various scenarios. The leaders also emphasized the importance of communicating simple nutrition messages and having cross-age peers that are representative of the community where they are working. Table 6 describes the interventions included in Group E.

### 3.6. Group F: Community, Afterschool or Extracurricular Interventions That Trained Same-Age Peers

Ten interventions in our review trained same-age peers to deliver nutrition education information outside a classroom context. These interventions were generally less structured than those that were classroom-based; only one had a weekly schedule of lessons that peers delivered [68]. A key theme across this group of interventions was having simple messaging that was easy for same-age peers to use in conversation, whether in an informal or more formal context.

Three interventions were informal in their structure: same-age peers were trained and then tasked with spreading the messages through their normal social networks [23,39,66]. Two of these were essentially the same intervention implemented in two different countries and focused on promoting water consumption [39,66]. Peers brought up water consumption in conversation and also modeled drinking water throughout the day. Of note, these interventions measured changes in SSB intake as well as in water intake, but the message was positively focused on water consumption rather than on decreasing SSB consumption.

Four of the interventions took place either during the school lunch period or after school. Same-age peers promoted healthy eating and conducted taste tests in the cafeteria [24,25,26], planned their own events with peers [65], or taught lessons and promoted nutrition messages through club activities [68]. The remaining three interventions involved a small group of the target population who participated in afterschool programs where nutrition was incorporated into another activity. Two of these interventions were photovoice projects, where youth took pictures in their neighborhoods that gave visual representation to the food environment and then showcased their work in a community exhibition [55,63]. In one of the photovoice projects, a student took her photographs of the school cafeteria food to the food service director and successfully advocated for healthier options [63]. The third of these types of interventions was an afterschool theater program in which youth wrote a play about healthy eating and performed it in a dinner theater setting for their friends and family [48]. These interventions represent creative ways for youth to connect nutrition with other hobbies or interests and to use their voices to advocate for the health of their community. The interventions in Group F are further described in Table 7.

## 4. Discussion

This review synthesizes components of nutrition education interventions targeting middle school-age students across a wide range of settings and program designs, all of which incorporate some version of a train-the-trainer model. The use of a framework synthesis approach to organize the interventions allowed for key themes to emerge from various settings that can inform future nutrition education interventions that employ train-the-trainer methodology and target the middle school population, especially in low-resource, urban settings. By focusing on training components, intervention content and design, and process evaluation results rather than primarily on outcomes [10,11], this review makes a unique contribution to the literature on youth nutrition education interventions.

Our first dimension for synthesis of themes centered on train-the trainer approaches, and we found that engaging sessions that provide opportunities for role play and talking through scenarios that may arise with the target youth were well-received [49,50,51,56,57,70,72,73], especially when those being trained were cross-age or same-age peers [24,25,38,65]. For example, the Foley et al. paper describes trainers interactively teaching the curriculum to cross-age peers and then giving the youth the opportunity to practice teaching the lessons during the training [38]. Additionally, providing follow-up training sessions and/or regular contact with intervention facilitators to provide support and troubleshoot can help ensure intervention fidelity [28,29,30,31,32,45,46,47,51,65,70]. Training sessions also included emphasis on adapting interventions to the local context and using culturally relevant examples [49,50,52,53,69,71]. The interventions included here underscore the importance of mentors and role-models who go beyond teaching nutrition information to modeling healthy behaviors themselves, which has been identified previously in the literature [74,75]. Additionally, when interventions engage trainers and allow them to connect their interests—whether in sports, gardening, or the arts—to nutrition, the youth receiving the intervention will also be more engaged [48,52,53].

Our second dimension focused on the intervention content. We found fruit and vegetable consumption [24,25,26,34,37,48,49,50,52,53,54,60,68,69], healthy beverages [23,33,34,39,40,41,42,43,44,46,47,59,61,66], and healthy breakfast [23,33,38,40,45,48,49,50,52,53,54,61,68] to be common topics. Beyond basic nutrition information, the interventions in this review commonly included goal setting [38,49,50,51,60], building skills to identify healthy options (including reading food labels) [52,53,54,59,60], and developing strategies to make healthy choices through an awareness of the food environment context [40,41,42,43,44,55,58,62,63]. Interventions included in this review also indicated that youth can be change agents in their communities and advocate for healthier food options in their school and neighborhoods [55,58,63]. In Leung et al., the intervention aimed to understand youth perceptions of food justice in their community using photovoice and equip them to promote positive change in their community food environment [55].

The third dimension focused on the format and delivery of the interventions, which as might be expected, varied by setting. Classroom based interventions followed a more structured curriculum, as did some afterschool programs, while other interventions relied on informal conversations among peers to disseminate information. When peers were delivering interventions, less structure sometimes led to less consistency [40,65]. One of the key findings within this theme was creating nutrition messages that are positive and simple, especially if the intervention is delivered in a less structured context outside the classroom [39,41,42,43,44,66]. Smit et al. and Franken et al. demonstrated this with their water promotion intervention implemented in the Netherlands and Aruba, respectively. The main intervention message was framed positively, encouraging water consumption, rather than discouraging sugar-sweetened beverage consumption, yet participants still reported significant decreases in their intake of sugar-sweetened beverages [39,66].

Our fourth and fifth dimensions synthesized outcomes and feedback from the youth participating in the intervention and from those trained to deliver the intervention, when measured. We were particularly interested in youth engagement, receptivity, and feedback provided through surveys and focus groups. Participant engagement and receptivity were measured through a wide range of approaches, when they were actually included as an evaluation metric. Only three interventions explicitly mentioned using student satisfaction surveys [28,29,30,31,32,33,52,53], and all three used different scales. Other interventions obtained feedback from those delivering the intervention or observers about youth engagement [28,29,30,31,32,41,42,43,44,64], making it difficult to compare receptivity across interventions. Focus groups were helpful in identifying which nutrition messages were most commonly retained, the components of the interventions that participants most enjoyed, and barriers to making dietary changes. For example, both Luesse et al. and Molaison et al. heard from youth that the lack of availability of healthy foods at home and in their communities made it difficult to make healthy choices [60,62]. Feedback from those delivering the intervention was reported less often, but both Foley et al. and Gittlesohn et al. obtained feedback from the cross-age peers who delivered the intervention and found that the older youth were implementing what they were teaching in their own lives [38,41,42,43,44]. We extracted data on the types of outcomes that were measured in the interventions, which commonly included anthropometric data, dietary intake, and knowledge, attitudes and behaviors related to healthy eating, but we did not focus on the results of these outcome measures themselves.

While evaluation of intervention efficacy is undeniably essential, improving youth health outcomes is a long-term objective, making it difficult to quantify success in the short-term with anthropometric measurements or behavioral questionnaires. Although BMI is well-correlated with fat mass and percent body fat in youth, it can be difficult to discern whether BMI reductions post-intervention actually indicate a reduction in adiposity, the more important result for improving health outcomes [76,77]. Additionally, changes in BMI may not necessarily reflect dietary shifts, as a number of factors, including physical activity and developmental changes, also have an effect on BMI. Furthermore, dietary assessment methods for youth yield imprecise information, and validity tends to be lower for food frequency questionnaires—the type of evaluation tool favored in the interventions included in this review due to their lower administrative burden [78]. Where resources are not available to measure long-term changes in dietary behaviors and outcomes such as body composition or biochemical markers of chronic disease risk, we posit that information about youth and trainer engagement, such as the content extracted in this review, provides good indication of whether an intervention will influence the habits of youth, leading to improved nutrition outcomes and health over the long term.

### 4.1. Limitations

One of the major limitations of this review was the variability in process and outcome measures used to evaluate the efficacy of the interventions. Such variability, as well as the limitations of outcome evaluation measures described above, make it difficult to determine which methodologies or designs would be most efficacious for training those delivering the intervention and for improving youth nutrition outcomes. The variability in process measures also limited our ability to assess youth acceptability of the interventions in a systematic way, though we were still able to identify components that were particularly engaging for youth across multiple interventions. The review was also limited in that few of the studies examined the effect of the intervention on the “trainers,” which could serve as another important marker of long-term impact in creating a culture of health for youth. Finally, while including a wide range of study types was beneficial in the additional insight provided through qualitative methods and study protocols, it did limit the depth of our quality assessment

### 4.2. Application of Findings

We incorporated the major themes identified through this review into a nutrition training for the COACHES project, a sports-based youth development intervention targeting middle school students in New Orleans, Louisiana. In the initial training for the near-peer coaches working with the youth, we emphasized three key messages that were simple, positive, and focused: fruit and vegetable consumption, maintaining hydration-primarily through water intake, and eating breakfast regularly. These three messages were included in nearly every intervention in this study and are highly relevant for our target population, particularly due to their participation in sports [79,80]. In the initial training, we covered goal setting, as well as skills and strategies for making healthy choices, in the context of the three key messages. We communicated this content to the coaches through interactive games and activities that they could then use with their youth. We conducted a follow-up training four months after the initial training to address strategies for overcoming both internal (e.g., reluctance to share about food choices due to self-consciousness) and external (e.g., lack of affordable, healthy options in the food environment) barriers youth face when seeking to make healthful dietary choices, and we used role-playing activities to address scenarios that coaches encounter in their work.

## 5. Conclusions

This review provides important insights and enhances understanding of the design, methodology, and structure of train-the-trainer nutrition education interventions for middle school youth in U.S. and U.S.-comparable urban settings. Several of the studies included here also illuminate barriers that youth face to healthy eating, especially in urban environments with limited affordable, fresh food options and an abundance of inexpensive, high-calorie processed foods. In middle school, youth have increasing, though not complete, agency over their food choices, and conversation about the food environment at home, at school, and in the broader community should be included in nutrition education interventions, as should devising strategies to navigate those food environments in healthy ways. One of the limitations of this review was the lack of consistent methods for evaluating youth receptivity of the intervention. Further research should include more measures of engagement and youth feedback, not only immediately post-intervention, but also after several months post intervention to determine whether messages have stayed with the youth and continue to motivate healthy behavior change.

## Figures and Tables

**Table 1:** Framework for categorizing nutrition education interventions.

		Population Trained		
		Adults	Cross-Age Peers	Same-Age Peers
Intervention Setting	Classroom (part of school curriculum)	A	B	C
	Afterschool, extracurricular, or school club activities occurring outside the classroom	D	E	F

**Table 2:** Group A: Classroom-based interventions that trained adults.

Author, Year	Intervention Name or Description	Population Characteristics: Grade Level/Age (Sample Size), Location	Group Receiving Training	Training Design	Intervention Frequency and Duration	Evaluation Indicators and Notable Results
Arlinghaus, 2017 ^1^ [21]	Obesity prevention program with compañeros	6th and 7th grade (*n* = 506), Houston, TX	PE teachers	Teachers were trained in leading all aspects of the intervention, which focused on basic physical activity and nutrition education. They were provided with strategies for using positive reinforcement and constructive feedback with students. Those who worked with cross-age peers (see Group B) met with them regularly to provide feedback and guidance. Teachers met weekly with research staff to discuss any issues.	Intervention was delivered during middle school students’ regularly scheduled PE class period for 6 months; 50 min a day, 5 days a week. One day each week focused specifically on healthy eating.	Outcome: change in anthropometrics; only students with BMI percentile at or above 85% at baseline (*n* = 189) were included in the analysis. Process: fidelity of implementation and a random assessment of 10% of classes to record frequency of positive reinforcement and constructive feedback.
Baskin, 2009 [22]	Described design, implementation and lessons learned from a pilot obesity prevention program implemented in a low-resource school	7th and 8th grade (*n* = 113), Southern U.S.	PE teachers, health educator, and graduate students who served as teacher aids during lessons	Initial training provided when curriculum was finalized. Training emphasized active learning and hands-on activities. Researchers met quarterly with those implementing the intervention to address any issues that arose and discuss any program modifications.	Intervention was implemented over one school year. Students had a health education period once a week during their normally scheduled 50-min PE class. The curriculum included food demonstrations and tastings.	Outcome: changes in anthropometrics, health behaviors, dietary intake, and physical fitness. Process: meetings with school staff and research meetings were recorded and analyzed, and themes were identified. Focus groups were conducted with students and staff to determine acceptability. Results: In focus groups, students reported making changes to their diet and thought the program should continue and be expanded to 6th graders. They enjoyed interactive games but reported hesitancy to try new foods and dislike of “healthier” foods in the school cafeteria.
Bukhari, 2011 [27]	Diet for a Healthy Planet with Teen Battle Chefs curriculum	9th grade (*n* = 98), Brooklyn, NY	Classroom teachers	2-day training focusing on the skills necessary to teach program lesson plans. During program implementation, teachers completed an online lesson feedback survey to provide information about fidelity and feasibility.	19-week program addressing state educational standards for high school and intermediate school. Offered as an elective at the school for a semester; class was daily for 1 h.	Outcome: changes in dietary behaviors, attitudes, and frequency of eating meals with friends and family. Results: Participants reported eating vegetables as snacks significantly more often post-intervention compared with a control group. Process: reach, focus groups with students, reflective exercises for students, dose delivered, and feasibility and fidelity as reported by teachers. Results: In focus groups, students reported eating more fruits and vegetables and an increased willingness to try new foods.
Contento, 2010 [28] +4 Papers [29,30,31,32]	Choice, Control and Change	7th grade (*n* = 278 in pilot; *n* = 1136 in RCT), New York, NY	Science teachers	A 3-h intensive professional development session was held for teachers prior to the intervention start, and a follow-up 3-h training session was held in the middle of the intervention. A research staff member also attended one-third of all classroom sessions and met with each teacher weekly to provide support and feedback.	Curriculum consists of 24, 45-min, science and nutrition education lessons taught over 8–10 weeks. Some lessons were taught over multiple days for a total of 30–35 sessions. Curriculum aligns with national science standards.	Outcome: changes in food and activity choices; mediating variables measured included self-efficacy, outcome expectations, beliefs, attitudes, and perceived barriers. Results: Participants significantly decreased number of SSBs consumed and the portion size of SSBs compared to a control group. The pilot group additionally reported a significant increase in fruit and vegetable consumption, but the RCT intervention group did not. Process: teacher professional development, teacher implementation and student reception. Results: Student engagement level was evaluated by research staff during classroom sessions and the mean was 72%. Students were particularly engaged in hands-on activities. Mean student satisfaction with the program measured by survey was 2.9 on a 4-point scale.
Dubuy, 2014 ^2^ [33]	Health Scores!	10–14 years old (*n* = 605), Flanders, Belgium	Teachers	Prior to the intervention, teachers at participating schools received training on a range of activities related to healthy eating and physical activity. Further details about the teacher training and in-school curriculum were not provided in the paper. The key intervention strategy was using professional football players as credible role models for healthy lifestyle behaviors (see Group D).	4 month in-school curriculum bookended at the start and end by clinics with a professional football team	Outcome: changes in dietary habits, frequency of breakfast consumption, eating attitudes and self-efficacy, and physical activity levels. Results: Those in the intervention group increased water and vegetable consumption, and this trended toward significance compared with the control group. Process: youth satisfaction with professional athlete clinics, response to videos and letters and overall satisfaction with the program. Results: Youth overall satisfaction with the intervention was 7.82 out of 10, and the two intervention topics most commonly recalled by youth when asked were breakfast and vegetables.
Duncan, 2019 [34]	Healthy Homework program	7–10 years old (*n* = 675), Auckland and Dunedin, New Zealand	Classroom teachers at intervention schools and a lead teacher from each control school (who were permitted to implement the program after the final follow-up of the study)	Teachers received a half-day of training. 90 min was spent on the benefits of physical activity and healthy eating for students and previous strategies to integrate these topics into the curriculum. The second 90 min was spent on the program modules, examples of implementation and an opportunity for questions about the program.	8-week in-class and applied compulsory homework teaching module (length of a school term in New Zealand) grounded in the national school curriculum. The in-class portion was delivered in 3 90-min sessions a week, and one session included a review of the previous week’s homework. An online portal allowed students to share homework-related information with other students, including those at other schools.	Outcome: changes in physical activity, dietary patterns, screen time, and anthropometrics. Results: Participants had a significant increase in fruit consumption post-intervention compared with a control group, but the change was not sustained at the 6-month follow up. Anecdotally, students seemed more engaged in the physical activity topics that nutrition ones during the intervention.
Fahlman, 2008 [36]	Michigan Model “What’s Food Got to Do With It?” curriculum	Middle school (*n* = 783), Large metropolitan area in Michigan	Classroom teachers	8 h of in-service training was held on the curriculum and the textbook, “What’s Food Got to Do With It?”	8-session nutrition module that is part of larger statewide health curriculum for middle school taught over the course of 1 month.	Outcome: changes in dietary intake, nutrition knowledge, and healthy eating efficacy expectations. Results: Participants significantly increased consumption of fruits and vegetables post-intervention compared with a control group. Of note, about one-third of the initial sample was lost to follow-up.
Fairclough, 2013 [37]	Children’s Health, Activity and Nutrition: Get Educated! (CHANGE!)	10–11 years old (*n* = 318), Wigan, England	Year 6 Teachers	A 4-h training was provided to teachers in delivering the curriculum.	20-week curriculum with weekly 60-minue classroom sessions that were aligned with the national primary school curriculum. Also included homework tasks.	Outcome: changes in anthropometrics, physical activity, sedentary time, and food intake.
Heo, 2018 ^3^ [45]	HealthCorps	High school (*n* = 832), New York, NY	Program coordinator at each school teaches lessons, oversees program activities, and serves as a mentor for students Extra- curricular activities also develop youth from the target population as peer leaders (see Group F)	Coordinators receive three weeks of HealthCorps training over the summer as well as a week of professional development over the winter break. Coordinators also have weekly check-ins with program supervisors and at least one site visit from the program supervisor during the school year.	10 classroom lessons are delivered as part of the curriculum. Were delivered over the course of a semester or school year, depending on the school. For students participating in program activities outside the classroom, total exposure could be up to 45 h over 36 weeks.	Outcome: changes in anthropometrics, knowledge, and health behavior. Results: Students participating in HealthCorps significantly increased fruit and vegetable intake; there was no change in the control group. Process: site visit by program supervisor to ensure fidelity.
Irwin, 2012 ^2^ [46] +1 paper [47]	Get Fit with the Grizzlies	4th and 5th grade (*n* = 888), Memphis, TN	PE teachers	Teachers attended a half-day training workshop on the 6-week curriculum and the support services that were available to them to implement the curriculum (included web support and special activities with the Memphis Grizzlies NBA team—see Group D). Teachers were also trained to administer the pre and post-tests for the intervention. Each teacher also received two one-on-one follow-up trainings at their school during the intervention.	6-week mini-unit incorporated into PE curriculum; one lesson taught per week. Players, dancers and/or the mascot for the Memphis Grizzlies visited the schools for an assembly, and there was a district-wide Get Fit with the Grizzlies Achievement Day at Grizzlies home arena at the end of the program.	Outcome: changes in knowledge, eating behaviors, and physical activity habits. Results: Daily fruit servings increased significantly from pre to post- intervention.
Kipping, 2014 [49] +1 paper [50]	Active for Life Year 5 (AFLY5) (Adapted from Planet Health and Eat Well, Keep Moving interventions in the U.S.)	9–10 years old (*n* = 2221), Bristol City and North Somerset, England	Year 5 classroom teachers and learning support assistants	The training was 8–9 h over the course of 1 day in a location away from school. During the training, the intervention rationale was explained, lessons and homework activities in the curriculum were discussed and taught by the trainers interactively. Teachers and assistants also had the opportunity to ask questions. At the training, teachers were given freedom to adapt materials to their style and the range of abilities for their students, but all of the knowledge and skills from the curriculum could be imparted.	The curriculum consisted of 16 in-class lessons with 10 parent-child interactive homework activities, delivered over a period of 6–7 months (two out of three school terms).	Outcome: changes in physical activity, screen time, food consumption, and anthropometrics. Results: Participants showed a significant decrease in energy drink consumption post-intervention compared with a control group.
Koch, 2019 [51]	Food, Health, & Choices	5th grade (*n* = 1159), New York, NY (North Manhattan and South Bronx)	Classroom teachers and graduate student “curriculum instructors”	Teachers received a stipend to attend a 6-h professional development workshop the week before school began. The workshop provided an overview of the intervention, and teachers had the opportunity to practice intervention activities. Curriculum instructors attended the professional development with the teachers, and had an additional 2-h training on the intervention, and weekly 2-h meetings with the research staff during the intervention. Classroom teachers and curriculum instructors co-taught the lessons.	23 lessons replacing 2 mandated units in the science curriculum, delivered over 10 months. September—November was 2 lessons per week, December to April was 1 lesson per month (due to standardized testing preparation), and May was 2 lessons per week.	Outcome: changes in anthropometrics, dietary intake, outcome expectations, self-efficacy, behavioral intention, habit strength, goal-setting skills, competence, and motivation. Results: A wellness component was delivered in some schools that included a policy around foods that could be served during classroom activities. Students who received only the wellness intervention showed a significant decrease in SSB intake, but those who received the curriculum only or the curriculum and wellness together did not.
Lepe, 2019 [53]	Empowering Urban School Children to Increase Fruit and Vegetable Consumption Through EFNEP-Enhanced PSE Interventions (EMPOWER)	5th grade (*n* = 312), Pawtucket, RI	School health teachers and EFNEP para- professional educators	Teachers were trained in the Fresh Fruit and Vegetable Program (FFVP) nutrition education curriculum by SNAP-Ed staff. EFENEP educators received 2 2-h training sessions on the Policy, Systems, and Environment (PSE) curriculum they would be delivering to students. Their training also included an in-depth orientation to process evaluation methods.	10 30-min PSE lessons to be delivered every other week by trained EFNEP staff, designed to augment the 8-week FFVP curriculum taught by classroom teachers.	Outcome: changes in knowledge and fruit and vegetable consumption. Process: fidelity, dose delivered, dose received, reach, and program perception. Results: In focus groups, students reported asking for more fruits and vegetables at home, and school staff affirmed growth in student empowerment. Mis- communication between teachers and EFNEP educators was identified as a barrier to implementation.
Li, 2010 [56] +1 paper [57]	The nutrition-based comprehensive intervention study on childhood obesity in China (NISCOC)	6–13 years old (*n* = 9867), Shanghai, Chongqing, Guangzhou, Jinan, Harbin, and Beijing, China	Teachers, classroom tutors, and health educators	A 2-day training was held for the school staff that would be implementing the intervention. The training covered integrating the program into the school curriculum and performing the intervention activities. Teachers practiced the lessons during the training to ensure understanding.	The intervention took place over 2 semesters and included 6 nutrition lectures that lasted a minimum of 40 min. A cartoon-style nutrition handbook was developed for the students that was to be used along with the lectures.	Outcome: changes in anthropometrics, glucose and lipid profiles, attitudes, knowledge, and nutrition practices.
Mihas, 2010 [61]	Vyronas Youth Regarding Obesity, Nutrition, and Attitudinal Styles (VYRONAS)	12–13 years old (*n* = 191), Athens, Greece	Home economics teachers	Teachers attended two 3-h training seminars to learn about the objectives of the program, their role in delivering the intervention, and the importance of incorporating health and nutrition into the curriculum.	12 1-h lessons were delivered in the classroom over a period of 12 weeks. Teachers were supervised by a health visitor or family doctor from the community when delivering the lessons. Two meetings were also held for parents providing information about healthy dietary habits for children.	Outcome: changes in anthropometrics and dietary intake. Results: Participants showed a significant increase in fruit consumption post-intervention compared with a control group, and this increase was sustained 12 months after the intervention.
Olivares, 2005 [64]	The program sought to design and validate appropriate nutrition education materials for Chilean primary school students and develop and validate a teacher training program that could be replicated throughout the country.	3rd—7th grade, (*n* = 1701), Chile (intervention was implemented in schools throughout the country)	Classroom teachers	Participants received 3 days of training on the newly developed curriculum, which included a textbook, teacher’s guide, and practical guides for students that accompanied each of the modules. Some teachers who were particularly motivated by the training spontaneously trained colleagues when they returned to their respective schools.	The lessons were implemented over a period of 5 months.	Outcome: changes in knowledge and dietary intake. Results: Fruit and vegetable intake increased significantly among 10–11-year-old girls compared to a matching control group. SSB intake increased significantly among 8–9-year-olds in both the intervention and control groups. Process: teacher feedback on program perception. Results: Students were most engaged in activities where they prepared healthy foods.
Tsai, 2009 [69]	TAKE 10!	K-6th grade (*n* = 840), Chicago, IL	Teachers and a full-time volunteer	The training oriented the teachers and volunteer to the curriculum, provided strategies for integrating it into their other lessons, and connected health with academic learning.	The intervention was implemented over the course of a school year. Content was adapted to be appropriate for each grade level. The program includes daily 10-min physical activity breaks in the classroom and incorporates nutrition information into these breaks.	Outcome: changes in anthropometrics and nutrition and physical activity knowledge. Process: program perception evaluated through observations of the sessions and interviews with teachers and students at the end of the intervention. Results: Teachers were generally positive about the program and implemented 3–5 of the sessions per week. Students reported better concentration after moving during the sessions and recalled nutrition information they learned in the post-intervention interviews.
Tucker, 2015 ^2^ [70]	Let’s Go 5–2-1–0 childhood obesity prevention intervention delivered by school nurses plus 1:1 or small group coaching by nursing students	4th and 5th grade (*n* = 72), Location not specified; corresponding author affiliated with University of Iowa	School nurses Intervention also included nursing students as mentors (see Group D)	Training for school nurses on delivering the classroom portion of the intervention was not described in the paper.	School nurses delivered the Let’s Go 5–2-1–0 curriculum weekly during classroom instructional time in 10–15 increments. Dosage varied from 14–21 sessions. Nursing students had lunch with their assigned student/students weekly to discuss curriculum content and set health goals.	Outcome: changes in anthropometrics, physical activity levels, nutrition, family eating patterns and screen time. Results: Self-reported daily servings of fruits and vegetables increased significantly at both schools.
Zhou, 2019 ^2^ [72] +1 paper [73]	Chinese Childhood Health, Activity and Motor Performance Study (Chinese CHAMPS) with both in-school and afterschool components	7th grade (*n* = 680), Beijing, Wuhu, and Weifang, China	PE teachers Intervention also included afterschool program staff (see Group D)	2-day training focusing on adolescent growth and development, designing age-appropriate physical activities, and instructional methods. Training facilitators demonstrated activities, and staff then had the opportunity to practice.	8-month intervention; the in-school portion increased PE class to 3 days/week and implemented daily recess. As part of PE, students received fitness and nutrition education and bi-weekly text messages.	Outcome: Changes in physical fitness, anthropometrics, cognitive function, food habits and preferences, knowledge, physical activity Process: fidelity and dose received through periodic monitoring of sessions by research staff.

^1^ Also included in Group B. ^2^ Also included in Group D. ^3^ Also included in Group F.

**Table 3:** Group B: Classroom-based interventions that trained cross-age peers.

Author, Year	Intervention Name or Description	Population Characteristics: Grade Level/Age (Sample Size), Location	Group Receiving Training	Training Design	Intervention Frequency and Duration	Evaluation Indicators and Notable Results
Arlinghaus, 2017 ^1^ [21]	School-based obesity prevention program with compañeros	6th and 7th grade (*n* = 506), Houston, TX	High school students—“Compañeros” (based on the “promotoras” model of using peer health workers in the Hispanic community.	Cross-age peer leaders were trained daily for two weeks on intervention activities. In addition to the curriculum, they were trained to be able to identify strengths and weaknesses in their own eating habits and given ideas for how to engage in conversation with middle school students about healthy diet and activity habits. Training included opportunities to practice initiating conversations about the curriculum using different scenarios. They were also trained in providing praise and modeling healthy behavior. Throughout the intervention, they discussed the topic of focus with the PE teacher (see Group A) prior to each class and received regular feedback and support from the PE teacher.	Intervention was delivered during middle school students’ regularly scheduled PE class period for 6 months; 50 min a day, 5 days a week. One day each week focused specifically on healthy eating.	Outcome: change in anthropometrics; only students with BMI percentile at or above 85% at baseline (*n* = 189) were included in the analysis. Process: fidelity of implementation and a random assessment of 10% of classes to record frequency of positive reinforcement and constructive feedback.
El Rayess, 2017 [35]	Mark, Set, Go!	5th and 6th grade (*n* = 954), Providence, RI	High school students in an experiential learning program that includes community internships	Cross-peers received an orientation and weekly training session. Training covered classroom management, small group teaching strategies, and a review of the material.	9-week program (frequency of lessons not specified)	Outcome: changes in knowledge, dietary behaviors, anthropometrics, and physical activity Results: There was a significant decrease in the percentage of students reporting drinking soft drinks and juice at least once a day. In subgroup analysis, there was a significant decrease in both categories for girls and for students with overweight or obesity.
Foley, 2017 [38]	Students As LifeStyle Activists (SALSA)	Year 8 (same as U.S. 8th grade) *Focus of the study was the cross-age peers; subject number for year 8 students not available. Western Sydney, Australia	Year 10 students (*n* = 415)	Cross-age peers were trained by university students from health and education disciplines who worked themselves had received educator training from the project staff. The Year 10 students were trained to deliver the intervention in a one-day workshop and were given a scripted manual to use as a guide for delivering the intervention. As part of the training, students practiced delivering the lessons in front of one another.	Four 70-min lessons delivered to a Year 8 class by a small group of 4–6 cross-age peer educators. Length of time between first and fourth lesson varied (mean 25 days +/- 15.9)	Outcomes for *cross-age peers*: changes in dietary behaviors, physical activity, screen time and intention to change. Results: At follow-up, a significantly higher proportion was meeting recommendations for daily fruit and vegetable intake and drinking less than 1 cup of sugary beverages daily. Process: acceptability among cross-age peers Results: 91% would recommend the program to their peers; goal setting, leadership and teaching emerged as important themes, and 42% discussed program themes at home.
Lo, 2008 ^2^ [59]	Fluids Used Effectively for Living (FUEL)	Grade 9 (*n* = 113), Saskatchewan, Canada	University students (worked with same-age peers from the target population, see Group C)	Two-week training intensive that oriented the cross-age peers to the content and also aimed to build teamwork among them. Cross-age peers also provided leadership for the same-age peers.	Six-week program, one 45-min session each week	Outcome: changes in beverage intake, knowledge, and attitudes. Results: Significant decrease in SSB intake post-intervention, sustained through 3-month follow up among group with cross-age and same-age peers (three other comparison groups without peer educators did not show significant change). Process: participant satisfaction with content and delivery of the program. Results: 71% enjoyed the intervention and 77% would suggest it to others.

^1^ Also included in Group A. ^2^ Also included in Group C.

**Table 4:** Group C: Classroom-based interventions that trained same-age peers.

Author, Year	Intervention Name or Description	Population Characteristics: Grade Level/Age (Sample Size), Location	Group Receiving Training	Training Design	Intervention Frequency and Duration	Evaluation Indicators and Notable Results
Lo, 2008 ^1^ [59]	Fluids Used Effectively for Living (FUEL)	Grade 9 (*n* = 113), Saskatchewan, Canada	Same-age, Grade 9 peers (worked with cross-age peers, see Group B)	Had their own two-week training intensive to orient them to the content and build teamwork. They were connected to cross-age peers for guidance.	Six-week program, one 45-min session each week	Outcome: changes in beverage intake, knowledge, and attitudes Process: participant satisfaction with content and delivery of the program. See Table 2 for results
Stock, 2007 [67]	Healthy Buddies	4th–7th grade (*n* = 199) British Columbia, Canada	Cross-age peers. In this case, the students in the target age range for the study were the older peers	Cross-age peers received a lesson from the intervention teacher once a week and then taught that lesson to their younger “buddies” that same week. Older students still developing leadership skills were paired with another older student with strong leadership ability.	21 weeks—the cross-age peers receive (45 min) and then deliver (30 min) a lesson once per week. Paired classes also had two 30-min structured physical activity sessions together weekly.	Outcome: changes in height, weight, blood pressure, and heart rate; 9-min run, knowledge of nutrition and physical activity, and eating and physical activity behaviors.

^1^ Also included in Group B.

**Table 5:** Group D: Community, afterschool, or extracurricular interventions that trained adults.

Author, Year	Intervention Name or Description	Population Characteristics: Grade Level/Age (Sample Size), Location	Group Receiving Training	Training Design	Intervention Frequency and Duration	Evaluation Indicators and Notable Results
Dubuy, 2014 ^1^ [33]	Health Scores!	10–14 years old (*n* = 605), Flanders, Belgium	Professional football players hosted a start clinic and end clinic prior to and at the conclusion of the classroom-based portion of the intervention (see Group A)	While the professional athletes did not receive formal training from the research staff, the football clubs were responsible for organizing the start and end clinics, and athlete promotion of healthy behaviors was a key part of the intervention. At the clinics, the professional athletes participated in activities with the youth that encouraged a healthy diet and physical activity and handed out lifestyle contracts that youth signed. Athletes also filmed two video messages that were shown to the youth at school and sent two letters reminding students of the importance of eating healthy and being active.	4 month in-school curriculum bookended by the start and end clinics with a professional football team	Outcome: changes in dietary habits, frequency of breakfast consumption, eating attitudes and self-efficacy, and physical activity levels. Process: youth satisfaction with professional athlete clinics, response to videos and letters and overall satisfaction with the program See Table 1 for results
Gittelsohn, 2013 ^2^ [40]	Baltimore Healthy Eating Zones	10–14 years old (*n* = 242), Baltimore, MD	Public health graduate students	2-day initial training with periodic booster sessions. Once trained, they visited rec centers and corner stores participating in the intervention at least weekly and offered cooking classes at rec centers. Also worked with cross-peer educators at each rec center site (see Group E).	Four phases, each 10 weeks long. Interventionists and cross-age peers were to hold one session per week at rec centers.	Process: reach and dose measured against implementation standards developed by the research team.
Irwin, 2012 ^1^ [46] +1 paper [47]	Get Fit with the Grizzlies	4th and 5th grade (*n* = 888), Memphis, TN	Memphis Grizzlies players and staff	While athletes did not receive formal training, they participated as role models in the intervention by making visits to assemblies in participating schools and hosting an achievement day for youth who completed the program.	6-week mini-unit incorporated into PE curriculum; one lesson taught per week. Players, dancers and/or the mascot for the Memphis Grizzlies visited the schools for an assembly, and there was a district-wide Get Fit with the Grizzlies Achievement Day at Grizzlies home arena at the end of the program.	Outcome: changes in knowledge, eating behaviors, and physical activity habits. See Table 1 for results
Kohlstatdt, 2016 ^2^ [52] +1 paper [53]	NutriBee	4th–7th grade (*n* = 179), New Mexico, Michigan, Maryland and Guam	School teachers and health professionals. Intervention was conducted in afterschool or weekend clubs, or in camp settings	Received 1–3 h of experiential in-person instruction designed to parallel the format of the intervention itself, including a culminating game show. Also received an instructor training manual and informational video. After completion of the intervention, instructors received a stipend and certificate	10 2-h modules delivered across varying time frames ranging from 4 days (in camp settings) to 1 month (club that met once/week)	Outcome: changes in dietary knowledge, intentions, outcome expectations, self-efficacy, and dietary intake. Results: Selection of dried and fresh fruit and bottled water increased, and consumption of sugary sports drinks decreased. Process: measured dose, fidelity, and acceptability. Results: Acceptability was based on appropriateness of difficulty and appropriateness of length. Scores ranged from 1.5 to 1.8 out of a maximum of 2.
Linton, 2014 [58]	Youth Engagement and Action for Health (YEAH!)	Age range was 9–22 years across all groups: 6 middle school, 6 high school, 8 community center groups and 1 church youth group (*n* = 136), San Diego, CA	Adult mentors/ leaders of youth groups in a variety of settings (after-school programs, community organizations, religious organizations) who are interested in nutrition or physical activity- related community advocacy projects.	Training is a half-day session that covers gathering necessary resources for the project, conducting a community assessment of factors related to healthy eating and/or active living, identifying community decision makers, and advocacy. Adult leaders receive the YEAH! manual as part of the training and have access to ongoing technical support from the Sand Diego County Childhood Obesity Initiative throughout their projects.	Groups are run independently, and length varies by project; average was 9 sessions over a 10-week period. With guidance from an adult mentor, youth groups assess their food and built environment, prioritize problems identified in the assessment, and develop and implement an action plan to advocate with decision makers for change in their community.	Outcome: changes in attitude, perception of control, self-efficacy, readiness to act as social change advocates and health behavior. Process: retention of youth throughout project, number of meetings with decision makers. Results: 73% of youth participants across all groups remained engaged throughout their group’s project. 19 of 20 groups in the evaluation had in-person meetings or presentations with decision makers. 11 groups reported changes in the community as a result of their work, and 4 reported pending changes.
Luesse, 2019 [60]	In Defense of Food afterschool curriculum	6th–8th grade (*n* = 32), New York, NY	Afterschool program teachers	Before the start of the intervention, afterschool teachers received a 2-h professional development training on the curriculum. Two weeks into the intervention, teachers received an additional 1-h follow-up training. They also had access to support as needed throughout the intervention period.	10 weeks, one 2-h afterschool session each week. The curriculum is divided into 3 units of 3 lessons each and a final culminating lesson.	Outcome: changes in dietary intake, outcome expectations, self-efficacy, self-regulation skills and autonomous motivation. Results: There was a significant increase in fruit and vegetable intake post- intervention. Qualitative: assessment of student understanding and ability to apply “food rules” from each lesson and semi-structured interviews with youth to better understand target outcomes. Results: Youth best understood “rules” related to drinking water and moderation. In interviews, youth noted that the social and physical environment made it difficult to decrease consumption of processed foods even when they desired to do so.
Molaison, 2005 [62]	Qualitative study that examined factors that mediate fruit and vegetable consumption among southern, low-income black adolescents to aid in planning an intervention	10–13 years old (*n* = 42), 2 counties in the lower Delta region of Mississippi	Graduate students and research staff who were of the same ethnic or racial background as the youth.	3-day workshop provided training in standardized focus group methods.	Focus groups were conducted with youth enrolled in the 5-week National Youth Sports Program. Groups were segmented by gender and age: 10–11, 12, and 13, for 6 groups total. Each focus group met once during the 5-week summer program session.	Qualitative: focus group transcripts were coded, and themes were identified. Results: Taste and method of preparation were major factors limiting fruit and vegetable consumption, especially for vegetables. Additional limiting factors were lack of availability of fruits and vegetables at home or in neighborhood stores and limited control over their food options. Most had family support for fruit and vegetable consumption, but not peer support.
Tucker, 2015 ^1^ [70]	Let’s Go 5-2–1-0 childhood obesity prevention intervention delivered by school nurses plus 1:1 or small group coaching by nursing students	4th and 5th grade (*n* = 72), Location not specified; corresponding author affiliated with University of Iowa	Nursing students Intervention also included classroom component with school nurses (see Group A)	Training was provided in the 5-2–1-0 curriculum, basic motivational interviewing (MI) principles for behavior change, and role modeling healthy behavior. Training included a didactic portion, role playing, and use of videos. At one nursing school, training consisted of an initial 4-h session followed by weekly sessions of practice and role plays. At the other nursing school, a 2-h training session was provided.	The intervention ran from September to April in one school and January to April in the other school. School nurses delivered the Let’s Go 5-2–1-0 curriculum weekly during classroom instructional time in 10–15 increments. Dosage varied from 14–21 sessions. Nursing students had lunch with their assigned student/students once a week to discuss curriculum content and set goals for intervention topics.	Outcome: changes in anthropometric measurements, physical activity levels, nutrition, family eating patterns, and screen time. See Table 1 for results
Wright, 2012 [71]	Kids Nutrition and Fitness	8–12 years old (*n* = 251), Los Angeles, CA	An advanced practice nurse, registered nurses, a physical education specialist, and community health workers (some of whom were parent volunteers)	Training took place in a full-day, in-person session that covered all intervention protocols, including culturally relevant information and examples for the nutrition education component. Retraining was provided on an as-needed basis if instructors were not following the intervention protocol during session observations.	6-week afterschool program with weekly 90-min sessions. Students were recruited and went through the program in waves of 14–28 at a time. Beyond the curriculum, the intervention also included school and community-level activities, including physical and mental health services through local clinics.	Outcome: changes in anthropometrics, dietary behaviors, food preferences, knowledge, and self-efficacy Results: Self-reported intake of fruit, vegetables and 100% fruit juice increased significantly at post-intervention and was sustained at 12-month follow-up. Process: intervention fidelity assessed by session observations, focus groups with parents.
Zhou, 2019 ^1^ [72] +1 paper [73]	Chinese Childhood Health, Activity and Motor Performance Study (Chinese CHAMPS) with both in-school and afterschool components	7th grade (*n* = 680), Beijing, Wuhu, and Weifang, China	Afterschool program staff Intervention also included classroom component with PE teachers (see Group A)	1-day training focusing on adolescent growth and development, designing age-appropriate physical activities, and instructional methods. Training facilitators demonstrated activities, and staff then had the opportunity to practice.	8-month intervention. The afterschool portion of the intervention added 45 min of moderate to vigorous physical activity (MVPA) 2 days a week, provided fitness and nutrition education, and bi-weekly text messages to students.	Outcome: Changes in physical fitness, anthropometrics, cognitive function, food habits and preferences, knowledge, and physical activity. Process: fidelity and dose received through periodic monitoring of sessions by research staff.

^1^ Also included in Group A. ^2^ Also included in Group E.

**Table 6:** Group E: Community, afterschool, or extracurricular interventions that trained cross-age peers.

Author, Year	Intervention Name or Description	Population Characteristics: Grade Level/Age (Sample Size), Location	Group Receiving Training	Training Design	Intervention Frequency and Duration	Evaluation Indicators and Notable Results
Gittelsohn, 2013 ^1^ [40]	Baltimore Healthy Eating Zones	10–14 years old (*n* = 242) Baltimore, MD	Cross-age peers, 13–18 years old. 2–3 applicants were selected by the director at each rec center site	Trained to assist interventionists (see Group D) in program delivery at rec centers prior to the start of the intervention and provided with a procedures manual. Cross-age peers were to accompany interventionists, assist in activities and communication with customers at corner stores and eventually be able to lead activities independently. Cross-age peers were provided with a $100 monthly honorarium.	Four phases, each 10 weeks long. Interventionists and cross-age peers were to hold one session per week at rec centers.	Process: reach and dose measured against implementation standards developed by the research team. Results: 58% of rec center visits by interventionists had a cross-age peer also present for the activity. Cross-age peers averaged 0.2 rec center visits per week or 2.1 per phase (Standard was 1 visit per week or 10 per phase).
Gittelsohn, 2014 [41] +3 papers [42,43,44]	B’More Healthy Communities for Kids	10–14 years old (*n* = 508), Baltimore, MD	Wave 1: College students (18–22 years) Wave 2: High school students (15–18 years)	Interested cross-age peers had to complete an application and interview. For those selected, 12 training sessions were held prior to the start of the intervention (27 h of training), and bi-weekly booster training sessions were held throughout the intervention. Trained cross-age peers were grouped into 3–5 to deliver the program at rec centers. College students were paid for completion of the training program and time teaching sessions at rec centers. High school students received service learning hours and small financial incentives. Curriculum provided to cross-age peers included ice breakers, a script of questions to use during discussions. Leaders were combined into groups of 3–5 to deliver the intervention in rec centers.	14 60-min sessions taught bi-weekly over 6 months	Outcomes for *cross-age peers* (n = 25): changes in anthropometrics, dietary intake, food behaviors, psychosocial factors, and leadership skills. Process: dose, reach, and fidelity; qualitative interviews with cross-age peers after Wave 1. Results: In open-ended comments, youth participants reported enjoying games and cooking lessons. Discussions were the most challenging element in terms of youth engagement. Level of interest in topics varied; using poster board for youth to write on topics increased engagement. In interviews, cross-age peers indicated more scenarios/role-playing during training would have been helpful, nutrition content for youth should not be overly complicated, and important that cross-age peers are representative of the community in which they are working.
Kohlstatdt, 2016 ^1^ [52] +1 paper [53]	NutriBee	4th–7th grade (*n* = 170), New Mexico, Michigan, Maryland and Guam	High school students	Cross-age peers worked with a subject matter expert coach to connect their interests with nutrition in the form of a project that would be part of the NutriBee intervention material. The projects and questions were incorporated into the curriculum as 1 of the 10 2-h modules of the program. Upon completion, cross-age peers received a stipend, certificate, and service learning credit.	10 2-h modules delivered across varying time frames ranging from 4 days (in camp settings) to 1 month (club that met once/week).	Outcome: changes in dietary knowledge, intentions, outcome expectations, self-efficacy, and dietary intake. See Table 4 for results.
Saez, 2018 ^2^ [65]	Peer intervention was carried out within the larger PRALIMAP-INÈS (Promotion de l’ALIMentation et l’Activité Physique-INEgalité de Santé)	Grade 9 (last year of middle school) and Grade 10 (first year of high school) (*n* = 32), Vosges, France	Cross-age peers (peer ambassadors) had participated in the program the previous year and were one year ahead of the target population in school (Intervention also used same-age peers; see Group F) To be eligible, peers had to have a demonstrated ability to control their weight and be of a similar socioeconomic background as the target population.	Peers received a 2-h training session at the start of the school year, in groups of 2–4 at a time or individually if grouping was not possible. During the training, cross-age peers considered the strengths they would bring as intervention facilitators. They then brainstormed activities they could lead and practiced role-playing various scenarios they might encounter. Following role-playing, they received feedback and debriefing tips. Cross-age peers discussed their planned activities with the program coordinator; financial support was available if needed for the activities. They had regular contact with the program coordinator throughout the school year via phone and text messages for follow-up and support and had a mid-year face-to-face meeting.	Target was for cross-age peers to plan and carry out four activities with their assigned small group of younger peers throughout the school year. The peer intervention was part of a larger health intervention in the school setting and was meant to be more informal in its approach, where activities could also be conducted outside of school.	Outcome: changes in physical activity, mental health, and perceived health and quality of life Process: satisfaction, perceived appropriateness, dose received, and practicality Results: A total of 5 cross-age peers were trained and remained active out of 20 potential facilitators who initially accepted the invitation, and 1 successfully organized and implemented an activity. Youth were significantly more likely to accept the peer intervention when offered by the peer rather than an adult professional. Interestingly, though some youth reported mistrust of peers and concerns about social exclusion. Facilitators had a high need for support from the program coordinator and despite addressing this topic in the training, struggled to come of up with ideas for activities. Training feedback indicated that more than 2 h was needed, and more practical ideas for activities and interpersonal skills training would be helpful.

^1^ Also included in Group D. ^2^ Also included in Group F.

**Table 7:** Group F: Community, afterschool or extracurricular interventions that trained same-age peers.

Author, Year	Intervention Name or Description	Population Characteristics: Grade Level/Age (Sample Size), Location	Group Receiving Training	Training Design	Intervention Frequency and Duration	Evaluation Indicators and Notable Results
Bell, 2017 [23]	Activity and Healthy Eating in ADolescence (AHEAD)	Year 8 (equivalent to U.S. 7th grade) (*n* = 99 in pilot, *n* = 928 in exploratory trial), Bristol, UK	Peers leaders from within the target population. In the pilot, 19% were trained as peer supporters and in the exploratory trial, 17% were trained. All Year 8 students filled out a nomination questionnaire and those with the most nominations were invited to a peer leader recruitment meeting.	Initial training for peer leaders was a two-day out-of-school event. Sessions used drama, food preparation, technology and games to deliver key nutrition and physical activity messages to peer leaders. Content supported school curriculum and national school health programs. Each peer leader received a diary to write about interactions with their peers; diaries included healthy challenges to encourage behavior change. Peer leaders walked to the training to promote active transport, and food and drinks served at the training were prepared on site, with the help of peer leaders when possible. Trainers included master’s degree students and professional health and well-being trainers. Four school-based follow-up sessions were held with peer leaders throughout the school year.	Peer leaders were tasked with informally diffusing the health messages they learned in the training and modeling healthy behavior over the course of the school year. Post-intervention behavioral questionnaires were used to assess the extent of the diffusion, and focus groups were conducted with non-peer leader intervention participants.	Outcome: changes in diet and physical activity behaviors. Results: Post-intervention, students ate significantly more servings of fruit a day and ate breakfast significantly more often than students from a comparable control group. Process: recruitment and retention rates for peer leaders, structured observation and evaluation of training sessions, post- intervention focus groups with both peer leaders and non-peer leader intervention participants. Results: Peer leaders responded especially well to hands-on activities, games and role-playing in the trainings. School-based trainings were less enjoyable to students than the initial trainings held offsite. About 1/3 of intervention participants were aware of having talked with a peer leader about healthy messages and reported that this encouraged them to increase healthy behaviors.
Bogart, 2016 [24] +2 papers [25,26]	Students for Nutrition and eXercise (SNaX)	7th grade (*n* = 399 in pilot, *n* = 2997 in RCT), Los Angeles, CA	Peer leaders from within the target population. 21% of 7th graders were trailed during the pilot and 23% were trained during the RCT. A different group of leaders was recruited each week and each leader was asked to also recruit a partner for lunchtime activities.	Peer leaders attended a training session where they were taught to discuss SNaX messages with peers and family using motivational interviewing (MI) techniques. During the training, they engaged in role-playing using MI. They then recruited a friend to co-facilitate two lunchtime educational sessions with them. Peer leader training sessions were conducted by bachelor’s level facilitators who were themselves trained by a Ph.D. level clinical psychologist (4 h on motivational interviewing) and a Ph.D. level public health researcher (20 h on intervention content).	5 weeks with two lunchtime sessions per week facilitated by peer leaders that could include taste tests and giving out of promotional items. Intervention also included school food environment changes and school-wide marketing of SNaX messages.	Outcome: changes in school lunch participation and cafeteria purchases, BMI classification change over time, attitudes about the cafeteria and water consumption, intentions to drink water, and water consumption. Results: Fruit servings significantly increased in intervention schools, and students also drank tap water significantly more often post-intervention compared with control schools. Process: researchers monitored peer leader training sessions for coverage of all session elements.
Franken, 2018 [39]	Share H_2_O (based on intervention conducted by Smit, 2016)	5th and 6th grade (*n* = 377), Aruba	Peer leaders from within the target population. The 15% of boys and 15% of girls with the most nominations from their classmates were invited to attend the training to become peer leaders.	Training was 90 min and held during school hours. Peer leaders learned about the benefits of water consumption, worked on formulating their own arguments for increasing water consumption, practiced strategies for promoting water consumption with their peers and were encouraged to set an example with their own water consumption. Peer leaders received follow-up training sessions in weeks two and five of the intervention.	Peer leaders were tasked with promoting water consumption to their peers through their social networks over a period of 8 weeks.	Outcome: changes in consumption of water and SSBs and behavioral intention around beverage consumption. Students were also asked how often their friends consumed water and how often their friends approved or disapproved of them consuming water. Results: Students in the intervention group consumed significantly less SSBs post-intervention compared with control group. Those with a high perception of their friends’ approval for drinking water consumed significantly more water.
Heo, 2018 ^1^ [45]	HealthCorps	High school (*n* = 832), New York, NY	Students from among the target population could elect to join the Youth Lead Action Research program or participate in facilitating lunchroom activities.	Paper does not include description of formalized training for same-age peers; leadership in healthy eating comes through participation in the program activities beyond the classroom. In Youth Lead Action Research, students learn research methods to identify health needs in their school or community and develop projects that address those needs. In the lunchroom, youth work alongside the program coordinator (see Group A) to share samples of healthy foods and engage with their peers on a monthly basis.	10 classroom lessons are delivered as part of the curriculum. Were delivered over the course of a semester or school year, depending on the school. For students participating in program activities outside the classroom, total exposure could be up to 45 h over a maximum 36 weeks.	Outcome: changes in anthropometrics, knowledge, and health behavior. Results: Students participating in HealthCorps significantly increased fruit and vegetable intake post-intervention; there was no change in fruit and vegetable consumption from pre to post-intervention in the comparison group. Process: site visit by program supervisor to ensure fidelity in curriculum delivery.
Jackson, 2010 [48]	Healthy 4 Life	6th–8th grade (*n* = 15) Urban area, southeastern GA	Intervention participants themselves developed a theater production that communicated nutrition and physical activity to friends and family.	Each session began with nutrition and physical activity topics, team-building activities introduced theater dynamics, students made a healthy recipe or snack after the theater activity and ended with a physical activity session.	6 weeks, 75-min afterschool sessions twice per week. Throughout the six weeks, students used what they learned about nutrition, physical activity, and theater to develop a play, Getting on Track, which they gave as a dinner theater performance for family and friends.	Changes in knowledge, food and physical activity choices, and behavioral norms. Results: Small sample size gave insufficient power to generate statistical significance, but more students reported choosing fruit over sweets post- intervention.
Leung, 2017 [55]	Intervention incorporated photovoice into a food justice curriculum for middle school youth.	6th–8th grade (*n* = 12) East Harlem, NY	Intervention participants themselves used photovoice to conduct a community food assessment and conduct a food justice project, then presented their photos and projects to the community.	Sessions covered photography and photovoice; the food system and environmental influences on food choices; a community food assessment photographing barriers and facilitators of eating healthy in the neighborhood; discussions of potential solutions to community food issues and roles youth could play in promoting change; and interviews of community members to understand their experience of the food environment. Throughout the intervention, facilitators reinforced the role of youth as researchers and active participants in changing their community.	24 weeks total. The first 10 weeks focused on photovoice and included 6 90-min sessions at an afterschool program and 3 photo assignments. In the second 14 weeks, youth developed and implemented a food justice project. A celebration and photo exhibit were held at the end of the 24 weeks where youth shared and discussed their photos and project with community members.	Qualitative interviews and subsequent analysis to identify themes. Results: Through photos and focus groups, participants: (1) expressed distrust of the food industry, (2) identified the abundance of low-cost, unhealthy foods and product placement as barriers to healthy choices, (3) noted the importance family influence on their food choices and also their potential to be change agents in their family, (4) expressed concern about the health of family members and their own health, and (5) identified strategies for improving the food environment.
Necheles, 2007 [63]	The Teen Photovoice Project	13–17 years old (*n* = 13), Los Angeles, CA	Intervention participants, all of whom were members of the Youth Advisory Board from the UCLA/RAND Center for Adolescent Health Promotion (the center), used photovoice and resulting themes to create health education messages that were then presented to the community.	Sessions covered photography and photovoice, qualitative research methods, sorting and selecting photos, creating narrative on photos, generating themes, generating ideas for health education products, planning for opening exhibit.	Over a period of five months, youth participated in 9 2-h afterschool sessions. In small groups, they selected a concept for a health education poster, photos to use on the poster and the text for communicating the health message. An opening exhibit for the posters was held at the California Science Center.	Outcomes of the project were the creation of the posters and the opening exhibit. Results: Following the project, youth encouraged the center to do more research on childhood obesity using youth participatory research methods. One student took the initiative to share photos of unhealthy food sold in the school cafeteria with the school foodservice director and advocated for healthier options.
Saez, 2018 ^2^ [65]	Peer intervention was carried out within the larger PRALIMAP-INÈS (Promotion de l’ALIMentation et l’Activité Physique-INEgalité de Santé)	Grade 9 (last year of middle school) and Grade 10 (first year of high school) (*n* = 32), Vosges, France	Same-age peers (peer entrepreneurs) were in the same year in school as the target population. (Intervention also used cross-age peers; see Group E) To be eligible, peers had to have a demonstrated ability to control their weight and be of a similar socioeconomic background as the target population.	Peers received a 2-h training session at the start of the school year, in groups of 2–4 at a time or individually if grouping was not possible. During the training, same-age peers considered the strengths they would bring as intervention facilitators. They then brainstormed activities they could lead and practiced role-playing various scenarios they might encounter. Following role-playing, they received feedback and debriefing tips. Same-age peers discussed their planned activities with the program coordinator; financial support was available if needed for the activities. They had regular contact with the program coordinator throughout the school year via phone and text messages for follow-up and support and had a mid-year face-to-face meeting.	Target was for cross-age peers to plan and carry out four activities with their assigned small group of younger peers throughout the school year. The peer intervention was part of a larger health intervention in the school setting and was meant to be more informal in its approach, where activities could also be conducted outside of school.	Outcome: physical activity, mental health, and perceived health and quality of life Results: A total of 7 cross-age peers were trained and remained active out of 36 potential facilitators who initially accepted the invitation, and 5 successfully organized and implemented at least one activity. For additional evaluation results see Table 5.
Smit, 2016 [66]	Share H_2_O	9–13 years old (*n* = 210), The Netherlands	Peer leaders from within the target population. The 15% of boys and 15% of girls with the most nominations from their classmates were invited to attend the training to become peer leaders.	Training was 90 min and held during school hours. Peer leaders learned about the benefits of water consumption, worked on formulating their own arguments for increasing water consumption, practiced strategies for promoting water consumption with their peers, and were encouraged to set an example with their own water consumption. Water consumption from reusable bottles was also connected to environmental health. During the training, peer leaders identified drinking water themselves and sharing the health and environmental benefits of water consumption as key messages. Follow-up training sessions were provided during weeks 2 and 4 of the intervention.	Peer leaders were tasked with promoting water consumption to their peers through their social networks over a period of 8 weeks.	Outcome: water and SSB consumption, intention to drink more water. Results: Students in the intervention group reported a significant increase in water consumption and a significant decrease in SSB consumption post-intervention compared to a control group.
Tamiru, 2016 [68]	A peer-led nutrition education intervention that included health promotion through school media and clubs, as well as school and community events	11–19 years old (*n* = 992), Jimma, Ethiopia	Peer leaders selected from among the target population	Peer leaders had a training session with the research team to learn about their role and responsibilities. Leaders were provided with health promotion materials to use in group education. Teachers held peer dialogue sessions throughout the intervention to support peer educators.	Over 8 months, peer leaders taught a group of fellow students via demonstration and role-playing in weekly sessions.	Outcome: changes in knowledge, attitude, dietary quality and variety, and anthropometric measurements. The study also assessed food insecurity and its relationship to dietary intake. Results: Youth from food secure households had significantly higher dietary diversity compared with those from food insecure households. More than 2/3 of students reported their food was selected by their parent/guardian as opposed to self- selected.

^1^ Also included in Group A. ^2^ Also included in Group E.

## Supplementary Materials

The following are available online at https://www.mdpi.com/article/10.3390/nu13082749/s1. Figure S1: Electronic search strategy for PubMed/Medline Database.
