# Supplementary material for: Themes in Train-the-Trainer Nutrition Education Interventions Targeting Middle School Students: A Systematic Review
Source: Nutrients. 2021 Aug 10;13(8):2749. doi: 10.3390/nu13082749 (PMC8398099; doi:10.3390/nu13082749)
Supplement: Supplementary file 1 [file nutrients-13-02749-s001.zip › nutrients-1274628-SI.pdf]

Figure S1: Electronic search strategy for PubMed/Medline Database.

Search ((((((middle school OR youth OR student* OR adolescen* OR preadolescen* OR teen* OR school-age OR junior high OR young adult* OR young people OR "adolescent"[Mesh] OR "child"[Mesh])) AND (((nutrition* or diet*) adj2 (communication* or education or intervention* or messag* or advice or promot* or aware* or behavi*)).tw. OR nutrition program OR obesity prevention OR food choice OR food preference* OR food consum* OR "Child Nutrition Sciences"[Mesh] OR "Nutritional Sciences/education"[Mesh] OR "Nutritional Sciences/methods"[Mesh] OR "Diet, Healthy"[Mesh] OR "Food"[Mesh] OR "Diet"[Mesh])) AND (coach OR mentor* OR mentor-led OR teacher OR peer OR peer-led OR role model OR participatory research OR "Peer Group"[Mesh] OR "Mentor"[Mesh])) AND (urban OR city OR poverty))) Sort by: Best Match Filters: Publication date from 2005/01/01 to 2020/05/28; English.
